# Exploiting biological and physical determinants of radiotherapy toxicity to individualize treatment

**DOI:** 10.1259/bjr.20150172

**Published:** 2015-06-17

**Authors:** J E Scaife, G C Barnett, D J Noble, R Jena, S J Thomas, C M L West, N G Burnet

**Affiliations:** ^1^ University of Cambridge Department of Oncology, Cambridge Biomedical Campus, Addenbrooke's Hospital, Cambridge, UK; ^2^ Cancer Research UK VoxTox Research Group, University of Cambridge Department of Oncology, Addenbrooke's Hospital, Cambridge, UK; ^3^ Oncology Centre, Addenbrooke's Hospital, Cambridge University Hospitals NHS Foundation Trust, Cambridge, UK; ^4^ Medical Physics Department, Addenbrooke's Hospital, Cambridge University Hospitals NHS Foundation Trust, Cambridge, UK; ^5^ Institute of Cancer Sciences, University of Manchester, Manchester Academic Health Science Centre, Christie Hospital, Manchester, UK

## Abstract

The recent advances in radiation delivery can improve tumour control probability (TCP) and reduce treatment-related toxicity. The use of intensity-modulated radiotherapy (IMRT) in particular can reduce normal tissue toxicity, an objective in its own right, and can allow safe dose escalation in selected cases. Ideally, IMRT should be combined with image guidance to verify the position of the target, since patients, target and organs at risk can move day to day. Daily image guidance scans can be used to identify the position of normal tissue structures and potentially to compute the daily delivered dose. Fundamentally, it is still the tolerance of the normal tissues that limits radiotherapy (RT) dose and therefore tumour control. However, the dose–response relationships for both tumour and normal tissues are relatively steep, meaning that small dose differences can translate into clinically relevant improvements. Differences exist between individuals in the severity of toxicity experienced for a given dose of RT. Some of this difference may be the result of differences between the planned dose and the accumulated dose (*D*_A_). However, some may be owing to intrinsic differences in radiosensitivity of the normal tissues between individuals. This field has been developing rapidly, with the demonstration of definite associations between genetic polymorphisms and variation in toxicity recently described. It might be possible to identify more resistant patients who would be suitable for dose escalation, as well as more sensitive patients for whom toxicity could be reduced or avoided. Daily differences in delivered dose have been investigated within the VoxTox research programme, using the rectum as an example organ at risk. In patients with prostate cancer receiving curative RT, considerable daily variation in rectal position and dose can be demonstrated, although the median position matches the planning scan well. Overall, in 10 patients, the mean difference between planned and accumulated rectal equivalent uniform doses was −2.7 Gy (5%), and a dose reduction was seen in 7 of the 10 cases. If dose escalation was performed to take rectal dose back to the planned level, this should increase the mean TCP (as biochemical progression-free survival) by 5%. Combining radiogenomics with individual estimates of *D*_A_ might identify almost half of patients undergoing radical RT who might benefit from either dose escalation, suggesting improved tumour cure or reduced toxicity or both.

Radiotherapy (RT) is the most effective non-surgical treatment of cancer.^1^ It is needed in the care of 50% of patients with cancer at some time in their illness, forms a major part of the treatment plan for 40% of those who are cured of their cancer and is primarily responsible for cure in 16%. Of those patients who receive RT, around 60% are treated with curative intent,^2^ so that radical RT is used in over 100,000 patients in the UK each year. The lifetime risk for cancer for people born since 1960 is now estimated to be over 50%,^3^ so that RT will be required for a quarter of the population at some point.

In terms of overall costs, cancer consumes about 5% of health spending, and of that, about 5% is committed to RT.^4^ Thus, RT is not only a highly efficacious treatment but also a highly cost-effective one.^1^ RT is an essential priority for the National Health Service to improve cancer survival to levels equivalent to those in countries with the best outcomes.^5^ Given the scale of use and value from RT, continuing investment in developing technologies is appropriate.^6^ This is all the more important as our population grows older, and cancers are diagnosed earlier. Developments in RT will also make an essential contribution to the Cancer Research UK vision of curing 75% of patients with cancer in 20 years' time.^7^

Intensity-modulated radiotherapy (IMRT) reduces dose-limiting toxicity. In turn, this has allowed dose escalation to improve local control and cure,^8–15^ so the issue of toxicity remains. Ideally, IMRT should be combined with image guidance to verify the position of the target. At its most interactive, image-guided radiotherapy (IGRT) uses daily CT imaging, on the treatment couch, to adjust the patient's position prior to treatment and to improve the accuracy of dose delivery.^16^ This provides an opportunity to use CT imaging for additional development work, including assessment of daily delivered (accumulated) dose (*D*_A_).

Major developments can be expected in RT, as the result of progress in numerous areas. One of these is the study of the genomics of radiation toxicity (radiogenomics), in which the UK is a leading contributor.^17,18^ Improvements in imaging for target volume delineation, treatment planning, technical developments in treatment delivery, developments in understanding of tumour response and ways to modify it resulting from genomics and imaging, molecular targeted RT, combination with pharmaceutical agents both old and new and drugs to abrogate toxicity represent some of the other areas of exciting research and development, which offer potential to improve the therapeutic ratio.^19–21^

This article reviews aspects of normal tissue toxicities considered from the point of view of both biological variation in (normal tissue) response and day-to-day variation in physical dose. The variation in toxicity that may be owing to underlying biological variation will be discussed. The calculation of accumulated dose, (*D*_A_), by recalculating the daily delivered dose based on image guidance scans will be addressed. Finally, the possibility of combining predictive testing of normal tissue sensitivity with estimates of *D*_A_ will be discussed.

## THE THERAPEUTIC RATIO

The success of RT in eradicating tumours depends on the total radiation dose delivered accurately. For most tumours, the higher the dose, the higher the chance of local tumour control and cure. There is a steep dose–cure relationship, both in experimental animal systems and in males. The fact that dose–response curves are steep is very important, since it indicates that small dose differences will translate into clinically relevant improvements. In most tumours, and animal systems, a 5% increase in dose will typically achieve an increase in tumour control in the range of 5–10%.^22^

However, there are limits to the RT dose that can be given safely, which are imposed by the tolerance of the normal tissues surrounding the tumour.^23^ As the dose is increased, so the incidence and severity of normal tissue damage also rises, and, when severe, normal tissue damage can produce significant morbidity that can be life threatening. Thus, selection of the appropriate treatment is based on a balance between lowering the dose to keep the incidence of severe normal tissue complications at an acceptably low level and raising the dose to increase the probability of local control and cure. Since toxicity impacts on quality of life and cancer survival rates are increasing, the avoidance of toxicity is growing in importance.

**Figure 1.**
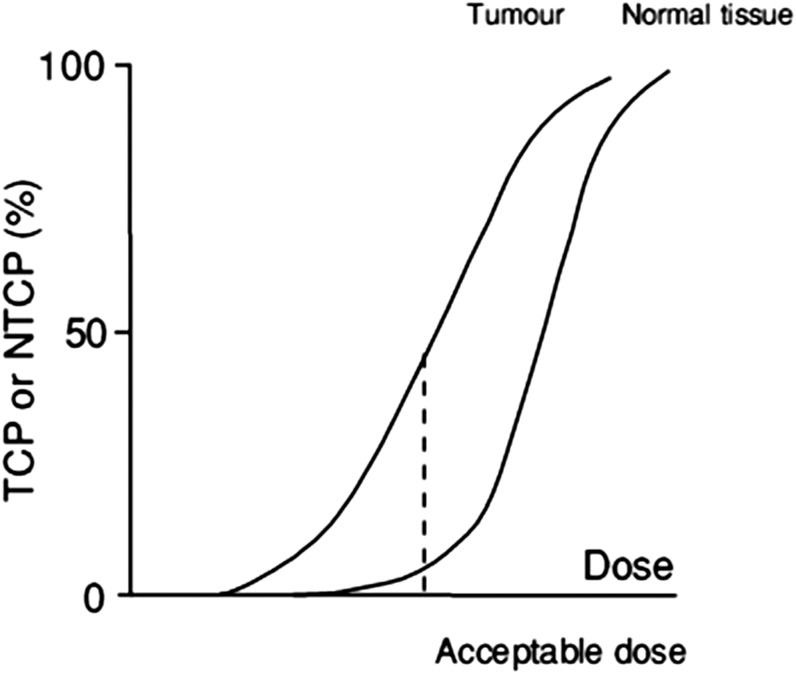
Sigmoid dose–response curves for tumour and normal tissue. A risk of significant normal tissue damage of 5% may be accepted in order to achieve a tumour control probability (TCP) of 50%, as illustrated here. For some tissues, such as the spinal cord, a 5% risk of late damage would be unacceptably high. The TCP curve can be shifted to the left by sensitising the tumour, for example with chemotherapy, although some agents may also have an effect on the normal tissue curve too. The normal tissue complication probability (NTCP) curve can be shifted to the right, for example using intensity-modulated radiotherapy and image guidance to spare more normal tissue from high doses. Ideally, strategies can be combined in order to shift the tumour control curve left and the normal tissue curve right. This will achieve a large rise in TCP with a fall in NTCP.

This balance between tumour control and potential toxicity defines the therapeutic window. This is often represented pictorially by classical sigmoid dose–response curves, and the size or “width” of the therapeutic index is represented by the distance between the curves for tumour control probability (TCP) and normal tissue complication probability (NTCP). In Figure 1, the standard therapeutic ratio is shown. This can be widened by adding strategies to sensitize the tumour, which shifts the TCP curve to the left, or protecting the normal tissue, which shifts the NTCP curve to the right.

In most circumstances, it is the “late-reacting” tissues that define tolerance, with the specifics depending upon the site treated. These toxicities typically do not improve over time and indeed may worsen. The breast represents an excellent example, in which modern RT techniques can improve outcome.^24^ However, some accelerated dose-fractionation schedules also lead to “acute” dose-limiting toxicity. One of the most dramatic is the continuous hyperfractionated accelerated radiotherapy) schedule for lung cancer, delivering 54 Gy in 36 fractions over 12 days.^25^ This highly effective schedule made ‘acute tolerance’ relevant. The addition of chemotherapy to RT, such as in head and neck cancer, also leads to acute toxicity that is close to dose limiting. However, late effects in these patients are also relevant in defining the upper limit of dose, even though some toxicities can be reduced by the use of IMRT.^12^ There is likely some relationship between the severity of acute and late toxicities in individual patients but not sufficient to use acute response as a predictor of late effects.^23^

### The steepness of the dose–response curves

**Figure 2.**
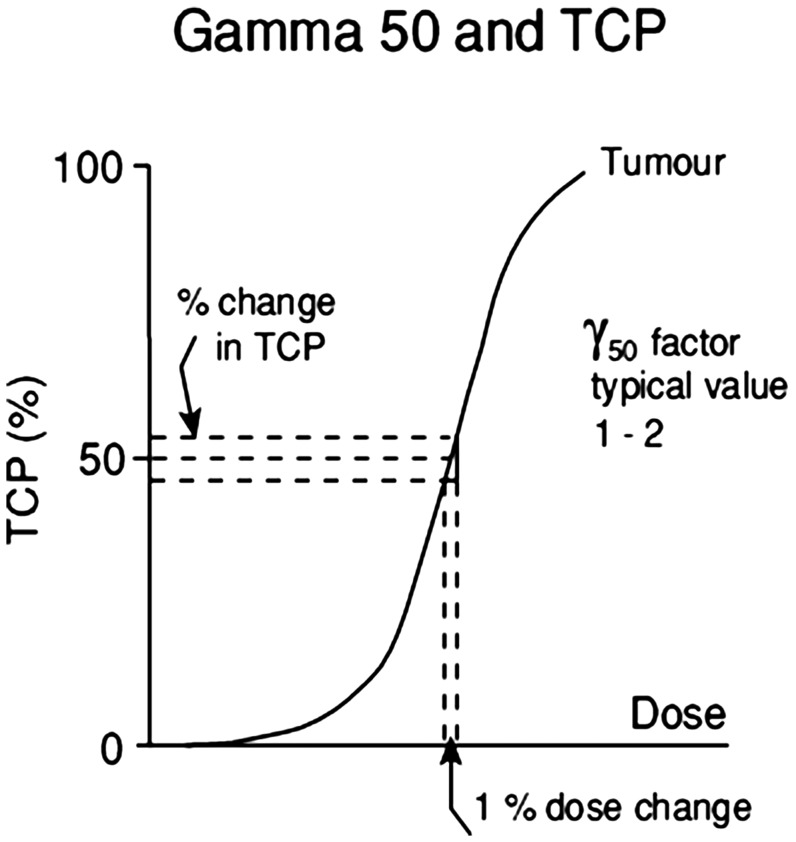
Diagram to illustrate the concept of the parameter γ-50, that is, the percentage increase in tumour control (or alternatively in normal tissue complication probability) for a 1% increase in dose at the 50% effect level. TCP, tumour control probability.

The steepness of the dose–response curve can be usefully described using the parameter Gamma-50 (γ_50_), which describes the percentage increase in tumour control for a 1% increase in dose, at the 50% TCP level (Figure 2). Thus, achieving a 5–10% improvement in TCP for a 5% increase in dose equates to a γ_50_ value of 1–2.

The γ_50_ concept can also be applied to normal tissues, although for many end points, the 50% NTCP level is rarely if ever reached. Here, it is usual to find γ_50_ values in the range 2–5, although some normal tissue dose–response curves may be shallower.^26,27^ Salivary gland toxicity, in the form of xerostomia, has a γ_50_ of 1,^28^ while the spinal cord has a higher γ_50_ of the order of 4.2,^29^ indicating that careful attention to small changes in dose, at near-tolerance levels, are even more important.

It is not always possible to *prove* that small dose changes improve outcomes.^22,30,31^ However, there is substantial evidence of dose response in both tumours and normal tissues, which provides proof-of-principle, and the value of small changes can be robustly inferred from these types of data. Indeed, it is important to do just that in considering opportunities for technological development. Small dose changes can be detected clinically, as demonstrated in the three-arm randomised trial with two experimental arms design,^32^ used, for example, in the START A^33^ and CHHiP trials.^34^

The slope of the sigmoid NTCP curves reflects heterogeneity between patients, which is considered to relate, at least in part, to normal genetic variation (that is, normal polymorphisms rather than rare deleterious mutations). In contrast to clinical findings, small animal experimental results show a steep dose–response curve. The steepness reflects lack of genetic variation between the inbred animals, highlighting a limitation of small animal data when studying normal tissue toxicities in humans.

## BIOLOGICAL DETERMINANTS OF RADIOTHERAPY TOXICITY

In a given treatment setting, different patients experience different severities of toxicity. Some of this variation is the result of differences in anatomy, of both the tumour target and surrounding normal tissues, leading to variation in the doses delivered to the normal tissues. A component of this dose variation results from day-to-day differences in position during the course of treatment. Factors involved include variation in patient positioning, internal organ movement or progressive weight loss during the treatment course. Positional variation can be improved by the use of IGRT.

Where the dose variation is minimized, additional variation is seen that is considered to reflect differences in underlying tissue radiosensitivity, and which, in turn, may have a genetic basis.^27,35^ Clinical evidence suggests that as much as 80% of variation in normal tissue response or toxicity may be owing to such biological variation.^35,36^ Investigation of this genetic aspect requires the best possible knowledge and control of dose.^27,37^

The importance of toxicity to both patients and society is increasing, as cure rates rise because of earlier cancer detection and more effective treatment. The financial cost of managing late effects of cancer treatment in survivors is high. Reduction of toxicity in cancer survivors will enhance the quality of life and reduce the social and population burden from morbidity. Reducing toxicity will also allow development of protocols for both dose escalation and combination with conventional chemotherapy and newer molecular-targeted agents.

As well as identifying patients with increased normal tissue radiosensitivity, it is also vital to identify patients with more radioresistant tissues. This group of patients could in principle be dose escalated to increase local control and cure, without increasing their risk of toxicity.

### Early descriptions of variation in individual normal tissue response

**Figure 3.**
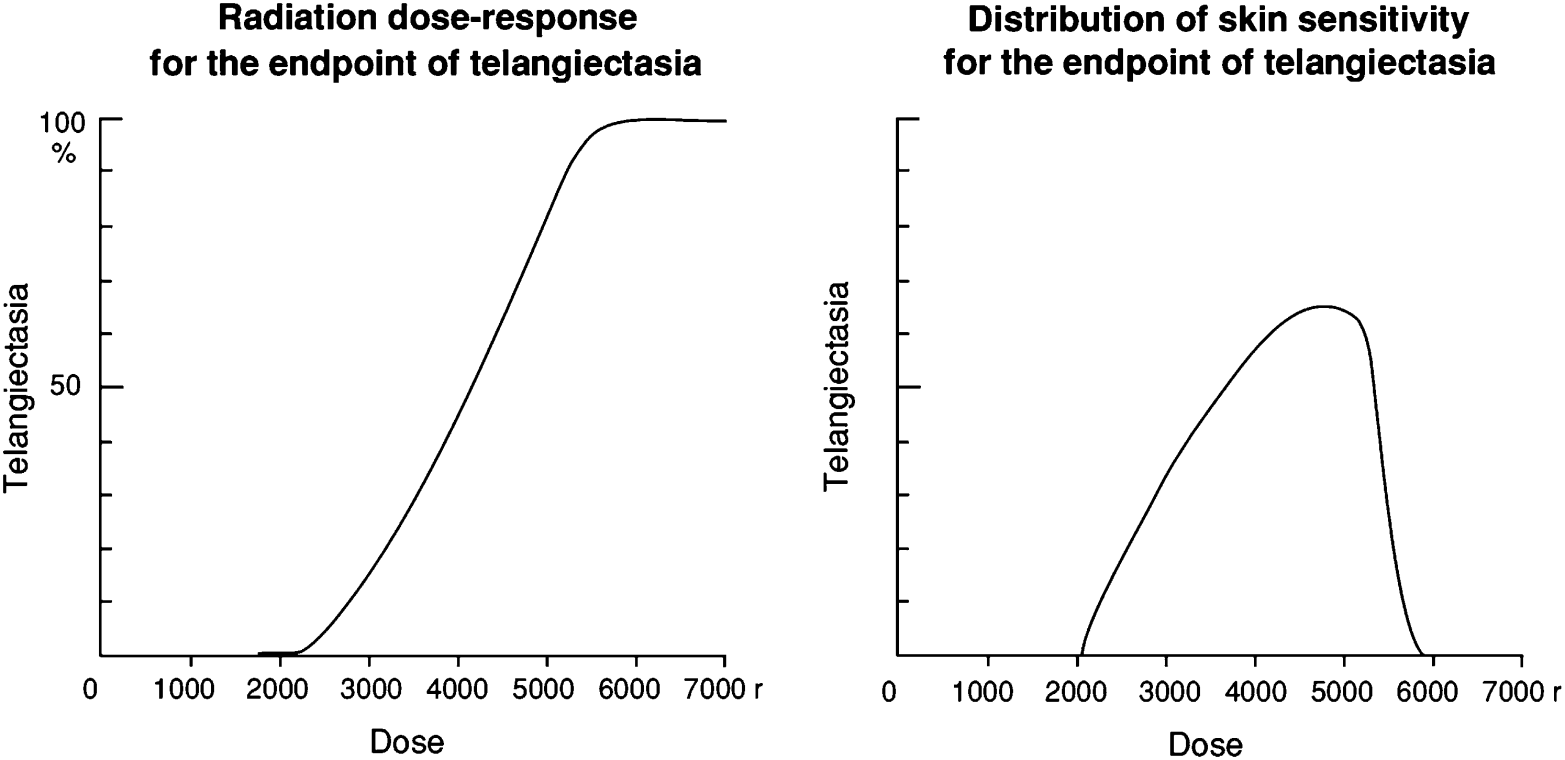
Classic sigmoid and bell-shaped dose–response curves for clinical normal tissue toxicity, redrawn from Holthusen.^38^ In this case, the end point was telangiectasia of the skin, and dose is measured in Röntgen (r): 100 r approximately equals 1 Gy. The sigmoid curve (left) represents a cumulative frequency distribution, whereas the bell-shaped curve (right), which has been created by transforming it, represents a differential frequency distribution. Note that this is not a true gaussian distribution because it has a finite range and is skewed. This is almost certainly a true representation of the biology, where at least extreme resistance to radiation is not plausible.

The sigmoid dose–response curve represents a cumulative frequency distribution, which is a transformation of a bell-shaped differential frequency distribution graph. Holthusen^38^ published the first formal description of this shape of dose response for *normal tissue* in 1936 (Figure 3), and his work is a seminal study in radiation oncology. However, the general notion of variation in normal tissue response between individuals treated with the same dose predates this. In the very earliest days of RT, at the beginning of the 20th century, dose was typically prescribed as the “Erythema Dose”, defined as the dose (or exposure, often represented by the time for which the X-ray tube was operating) required to produce erythema in 80% of the patients.^39^ This fascinating “unit” of dose implicitly embodies the concept of individual variation. This concept appears to have been lost, perhaps overshadowed by the introduction of objective physical measurement of dose based on ionisation, together with the introduction of megavoltage (MV) machines with skin-sparing beams.^37^

Individual variation with the classic bell-shaped frequency distribution was described again in the 1950s.^40^ Acute^40^ and late^41^ effects showed considerable variation, with an approximately gaussian distribution for both types of reaction. However, the seminal work defining dose response, and quantifying both acute and late normal tissue effects in clinical fractionation experiments on skin over a 20-year period, was performed in Gothenburg by Turesson et al.^35,37,42–44^

**Figure 4.**
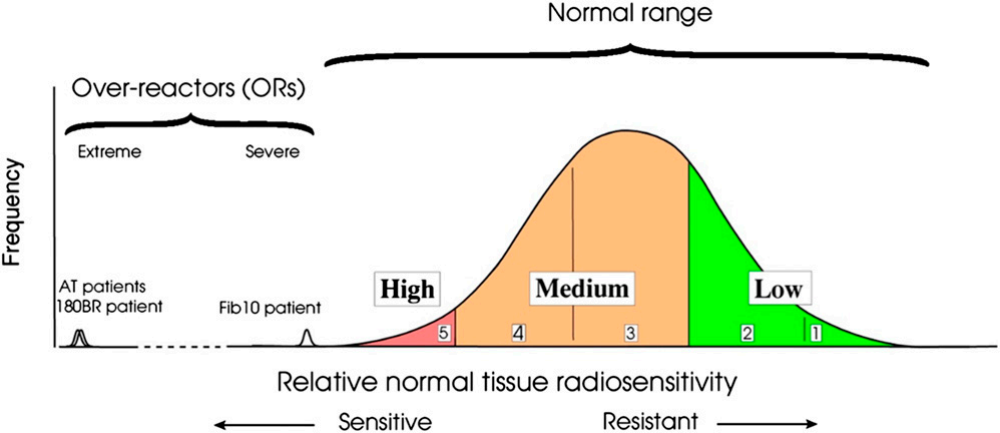
Stylized frequency distribution of normal cellular and tissue response shown on a relative scale. At the left, the sensitive end of the spectrum, some patients are known to have extreme sensitivity in both cells and tissues, including those homozygous for ataxia telangiectasia mutations. At least some of these patients (*e.g.* the patient whose cells were designated 180BR^45,46^) are also sensitive to chemotherapy agents with a mode of action involving DNA damage. Here, the normal range has been represented by an approximate gaussian distribution. Although this is not perfectly correct biologically, it can provide estimates for the standard deviation of the distribution. Questions remain about how far the tail extends to the sensitive (left) side of the curve; to the right, it is also likely that the distribution is truncated (Figure 3). The near gaussian shape is consistent with clinical data and also with cellular sensitivity data.^37,47^ It can reasonably be assumed that the range extends either side of the modal value for 2.5–4 standard deviations^37^ (adapted from Burnett et al^37^).

Several syndromes of extreme radiosensitivity are known and are typically associated with single genetic mutations, many in DNA damage response pathways, which render them sensitive. By contrast, variation in non-syndromic patients is thought to be polygenic, and the result of polymorphisms, that is normal variation, rather than deleterious mutations. The distribution of sensitivity can be represented by a theoretical gaussian distribution, with the syndromic patients shown as outliers (Figure 4).

The concept that individual variation in normal tissue response might be exploitable for predictive testing to individualize RT appeared in the late 1980s and early 1990s.^43,44,48–50^ An important step was the recognition that in addition to some individuals having greater toxicity, some have less normal tissue toxicity than average.^35,43,50,51^ This allows the possibility for dose escalation in more resistant patients, as well as for altered management in more sensitive individuals. Dividing patients into three groups, with 10% most sensitive, 50% intermediate and 40% more resistant, provides an opportunity to dose-escalate a large number of patients. Using data for late skin toxicity as an example, it might be possible to dose-escalate the resistant 40% by 17–19%.^23^ Even using a value of one for γ_50_, these dose increases should achieve an improvement in TCP of 17–19%. A theoretical link between intrinsic normal tissue and tumour radiosensitivity might appear to complicate this, but bigger gains are actually possible if they are correlated.^48,52^

As well as putative genetic variation as a cause for differences in toxicity between individuals, a number of potential modifying and confounding factors exist,^37,53^ such as use of other treatments (*e.g.* concurrent chemotherapy or surgery), medications,^54^ patient factors (*e.g.* age, smoking, comorbidities such as diabetes or hypertension and diseases such as scleroderma), dosimetric factors (radiation doses to normal tissues) or ethnicity, which need to be considered as co-variables when analysing genetic variation associated with RT toxicity. Surgical outcomes are also important. For example, in breast RT, a poor post-operative cosmesis is an important determinant of outcome.^24,55^ Prospective data collection is essential, and data on modifying/confounding factors are not always recorded well.

### How large is the biological variation in normal tissue response?

**Figure 5.**
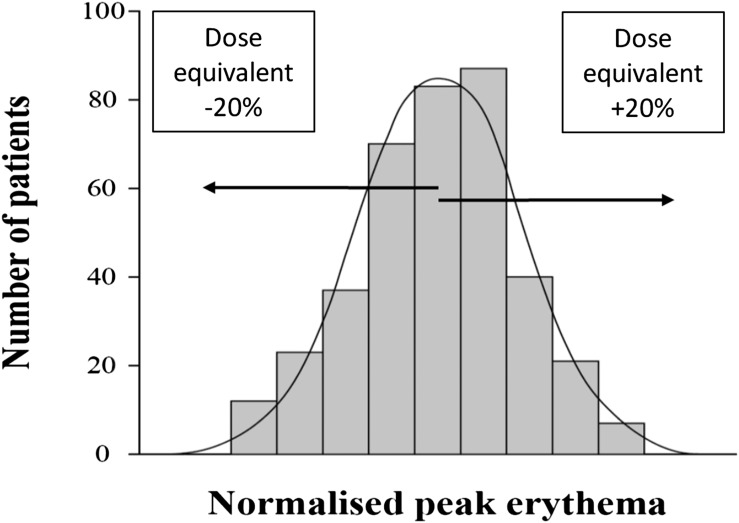
Frequency distribution of normalized peak erythema of the skin (acute reaction), with a near gaussian shape (redrawn from^37^). Superimposed is the estimate of the variation in dose required to move an “average” patient from the mean of the distribution to the sensitive or resistant ends (Turesson^37^). The variation of ±20% can be used to estimate the potential for dose escalation in resistant patients. It can also give an indication of the dose reduction required, or its equivalent achieved with, for example, hyperfractionation, to avoid toxicity in more sensitive patients. A similar dose equivalent range (±23%) has been observed in large studies of fibroblast cellular sensitivity in cells taken from normal patients having radiotherapy.^37,47^

A useful method of allowing comparison between biological variation and physical dose variation is to translate the range of biological variation into a dose equivalent. Using this approach, Turesson estimated that moving from the mean to either end of the bell-shaped distribution is equivalent to ±20% change in dose (Turesson, personal communication) (Figure 5).^37^ The variation of ±20%, which applies to both acute and late skin reactions, can be used to estimate the potential for dose escalation in resistant patients or the size of dose reduction required, or its equivalent, for sensitive patients.

A similar dose equivalent range (±23%) has been observed *in vitro* in large studies of fibroblast cellular sensitivity in cells taken from non-syndromic patients having RT.^37,47^

### Early efforts at predictive testing

In the late 1980s and early 1990s, radiobiologists investigated various approaches for measuring radiosensitivity to predict a patient's likelihood of developing RT toxicity.^18,56^ The mainstay of these investigations was the clonogenic survival assay. Most of the work was performed using fibroblasts derived from the skin. Several small studies each showed a correlation between cellular sensitivity and normal tissue response,^49,57–59^ but this could not be replicated in larger studies.^47^ The lymphocyte G2 assay played an important role in establishing an inherited component to radiosensitivity (see below). A number of other assays were also explored intensively, although without success in this area,^60^ and several decades would have to pass before molecular genetic techniques became available to examine the underlying genomic variation.

The single exception appears to be the T-lymphocyte apoptosis assay.^61^ Lymphocytes from 348 patients with a variety of cancers who experienced severe late RT reactions exhibited an impaired apoptotic response after 8 Gy *in vitro* irradiation.^61^ There was no relationship with acute reactions. There was also a reduced incidence of late toxicities in patients whose lymphocytes showed the greatest apoptotic response. The area under the receiver–operator characteristic curve for grade 3 late toxicity (which occurred in 8% of patients) was 0.92. The positive predictive value for grade 3 toxicity was 20%, and the negative predictive value was 98.5%. These results indicate considerable potential of the assay. This is undergoing further evaluation at present,^62^ and if its effectiveness is confirmed may revolutionize our approach to normal tissue radiosensitivity. However, at present, there is still no method for assessing radiosensitivity that can be used on a routine clinical basis.

#### Heritability of normal tissue sensitivity

It appears that radiosensitivity has a high heritability component, possibly accounting for as much as 70% of the variation.^63^ This is based on several studies that investigated lymphocyte radiosensitivity in patients with a range of reactions and in their first-degree relatives.^64–68^ The fact that heritability was demonstrated added to the concept that underlying genetic variation could be responsible for individual variation in toxicity.

#### Clinical individualisation of radiotherapy based on cellular radiosensitivity testing

During the era of clonogenic assays, there were important early efforts to address individualisation in some patients with radiation sensitivity syndromes. Patients with Fanconi anaemia (FA) had been known for many years to have increased sensitivity to DNA-damaging (clastogenic) agents, including cyclophosphamide and X-rays,^69–72^ related to increased chromosomal fragility. Despite increased DNA damage, there is evidence that cells from patients with FA have some DNA damage repair capability.^60,73^

One group was sufficiently disappointed with their results from bone marrow transplantation that they undertook clinical radiation sensitivity testing of their patients with FA on skin patches *in vivo* prior to the procedure.^74^ Although the testing was on individual patients, the results were combined to develop a standardized strategy of RT dose reduction, which likely did contribute to an improvement in outcomes.^72^ This work appears to be the first attempt to individualize RT doses based on a measurement of individual radiosensitivity, although in this case sensitivity was measured *in vivo* and applied to all patients with FA rather than individually. An enormous reduction in the administered dose of cyclophosphamide was also made and was clearly important in the modified schedule, so the exact contribution of the RT individualisation cannot be inferred.

In 1985, a patient with ataxia telangiectasia (AT) was treated with RT for medulloblastoma with a dose-reduced schedule, based on *in vitro* testing of his lymphocyte radiosensitivity compared with normal controls.^75^ Treatment was completed successfully, which is an important result given that cells from patients with AT are about three times as sensitive as normal cells for a given dose. This supports the hypothesis that intrinsic cellular radiosensitivity correlates with *in vivo* clinical normal tissue response to RT. It supports the concept that measurements of cellular sensitivity might be able to predict normal tissue response and so permit individualisation of RT.

#### Predictive molecular biomarkers

A molecular biomarker, TGF-b1, has been investigated as a predictor of radiation pneumonitis.^76^ This is a candidate molecule involved in fibrosis, although its exact role is not entirely resolved. However, despite early reports of a correlation,^76–78^ not all studies have demonstrated a relationship,^79^ and more work is certainly required.^80^

#### Candidate gene studies

Efforts to link specific variations in candidate genes, especially in DNA damage response pathways, to variation in toxicity were encouraged by the associations demonstrated for patients with radiosensitivity syndromes. For example, the genetic defect in a cell line (180BR) from a patient who developed severe chemotoxicity followed by fatal radiation toxicity^45^ was identified as affecting the function of ligase IV, a DNA damage response gene.^46^ Efforts to look at gene expression profiles, again in DNA double-strand break repair pathways, were also unsuccessful.^81^

Several studies did report associations between genetic variation in candidate genes and RT toxicity.^17,27,82–85^ There was initial excitement that, in a study of 41 patients, 7 alleles in 5 genes appeared to provide a “signature” of sensitivity for the development of subcutaneous fibrosis.^83^ However, all the candidate gene studies failed to validate.^86–88^ This may not be surprising, given that cancer susceptibility genes have been found in apparent “gene deserts”,^89^ which highlights that our understanding of the biology of cancer and its treatment is superficial at best. The possibility of linking a genetic signature with variations in physical dose has been suggested^85^ but still requires the genetic signature to be developed.

#### Genome-wide association studies

The development of technology to perform genome-wide association studies (GWASs) became available some years ago and has made a major contribution to the understanding of cancer risk.^89^ That technology has now been applied to radiogenomics. Logistically, there are some key issues in studying radiogenomics, including the need to wait for the radiation sensitivity “phenotype” (*i.e.* toxicity) to develop, which may take several years. It is also necessary to control for modifying/confounding factors, as noted above, and to collect data prospectively using validated toxicity scoring tools. Large numbers of patients (many thousands) are required for any GWAS, and this also applies to radiogenomics.

The last few years have started to produce definitive evidence that genetic variation is linked to toxicity.^88^ The first GWAS result to be reported was an association between a single-nucleotide polymorphism and the development of erectile dysfunction in African-American males after RT for prostate cancer.^90^ Further associations are emerging,^91,92^ and more are sure to follow,^93^ especially as a result of international collaboration, most notably based on the international Radiogenomics Consortium (RGC)^94^ and the REQUITE project.^95^ The RAPPER study,^18^ to which many UK centres have contributed patients, has made a major contribution to the RGC. Much more work, and especially much larger cohorts of patients, will be needed if we are to realize the holy grail of predictive testing. Nevertheless, the recent progress suggests that this will be achievable.

## PHYSICAL DETERMINANTS OF TOXICITY

Present RT treatments are based on static models of the patient anatomy and do not take into account variation in patient position or shape and location of mobile internal organs. Uncertainty in the dose actually delivered to normal tissues is recognized as a limitation in RT at the present time.^96^

### Considering accumulated dose—*D*_A_

Methods to calculate *D*_A_, as opposed to the planned dose, will provide a better understanding of dose–response relationships in normal tissues and allow development of active monitoring of delivered dose and predicted toxicity during the course of treatment. In turn, this will provide the opportunity to alter the treatment plan accordingly, and even small differences will be clinically worthwhile. The better the normal anatomical structures can be visualized, the easier this process will be, particularly since automated methods for contouring will be required. Any improvement in imaging quality will be valuable, and in due course there may be a role for online imaging using MR-Cobalt and MR-Linac machines.

The discrepancy between expected and observed toxicity for *D*_A_ should represent the difference in radiation sensitivity between individuals, hypothesized to result from underlying genetic factors, as discussed above. Recruitment to the RAPPER study of patients in whom *D*_A_ has been calculated would add a valuable cohort, where more of the individual variation should be attributable to the underlying biology. This emphasizes the importance of linking physics and biology and the need for an interdisciplinary approach to this work.

### Organs at risk—the rectum as an example of a critical structure

The rectum provides an example of a critical normal tissue structure, which in effect is dose limiting in RT for prostate cancer. It is subject to day-to-day variation, which is discussed here. There are also minute-to-minute changes that can occur during a treatment fraction; these are harder to evaluate and control.^97^ Many other normal tissue structures are also important but require further work.

The rectum and prostate are mobile internal structures that can move up to 2 cm in the anteroposterior direction relative to the pelvis, from one day to the next.^98^ It has been known for some time that rectal filling varies with time during treatment and that this can have important consequences. For example, de Crevoisier et al^99^ demonstrated that in patients receiving RT for prostate cancer, the incidence of biochemical failure, a surrogate for local failure, was significantly higher amongst those who had a dilated rectum (defined as a maximum cross-sectional area of ≥11.2 cm^2^) at the time of planning. The same effect has also been described by other groups.^100,101^

**Figure 6.**
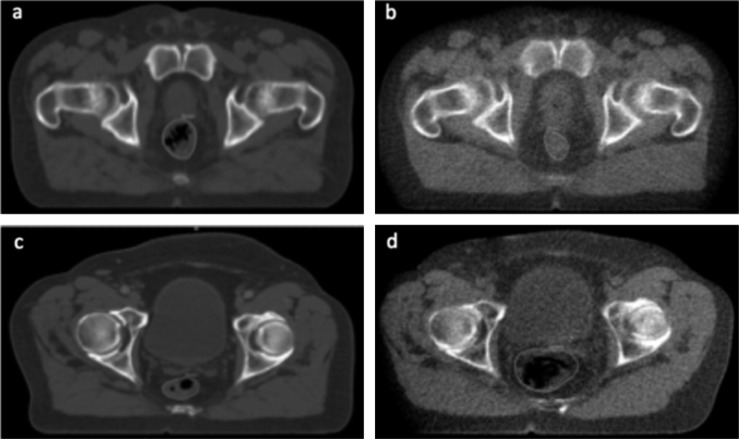
Axial slices at the same level from two patients to illustrate differences in mean position at treatment compared with that at planning. (a) Loaded rectum on kilo voltage (kV) scan from patient whose rectal position during treatment was 9.6 mm more posterior than at planning. (b) Empty rectum on Day 1 mega voltage (MV) scan from same patient as (a). (c) Empty rectum on kV scan from patient with rectal position during treatment was 3.2 mm more anterior than at planning. (d) Loaded rectum on Day 35 MV scan from same patient as (c). Reproduced from Scaife et al^102^ with permission from the British Institute of Radiology.

It is thought, very rationally, that this effect relates to movement of the rectum. A distended rectum can displace the prostate anteriorly. If this happens at the time of the planning scan but then resolves before treatment, the dose plan will underdose the posterior part of the target. This effect also alters the dose received by the rectum (Figure 6). Simple methods to control the size of the rectum include emptying the rectum before treatment,^103^ although this may not be straightforward in routine practice. Dietary interventions, a potential simple approach, have not been shown to alter the incidence and severity of gastrointestinal side effects.^98,104^

Toxicity can be modelled to a first approximation as a solid structure using the Lyman–Kutcher–Burman model.^105^ Although much of the existing dose–response data suggest that high doses are predominant in determining the risk of toxicity, there is also evidence of other subtle effects of dose. Analysis of the RT01 trial showed that the number of dose–volume histogram (DVH) points that were violated^106^ and the shape of the dose distribution were correlated with outcome.^107^

#### Variation in rectal position

An indirect way of studying possible changes in dose is to consider the position of the rectum during the course of the treatment, in relation to the treatment isocentre (or equivalent). A variety of studies have demonstrated rectal motion in patients treated with RT for prostate cancer.^108–115^ These studies were small and tended to rely on a limited number of images acquired during treatment, simply because of the logistics of collecting and contouring large numbers of scans.

Our VoxTox study has been set up to examine this in detail.^116^ VoxTox is an interdisciplinary research programme funded by Cancer Research UK, which brings together oncology, physics, engineering and mathematics.^116^ *D*_A_ will be correlated with toxicity in 2000 participants with cancers of the prostate or head and neck or tumours of the central nervous system in order to quantify the contribution of physical dose variation to toxicity. The basis of the imaging is the daily TomoTherapy® (Accuray, Sunnyvale, CA) MV fan-beam CT image guidance scans, which are performed on every patient every day.^117^

So far, scans from 10 patients treated to 74 Gy in 37 fractions (Figure 6) have been analysed, providing a total of 3900 slices; all were contoured manually by a single operator (JES).^102^ As expected, manual contouring was time consuming, with an estimated 12 h required per patient. This demonstrates that large-scale use of this imaging is impossible without automated contouring. Intraobserver variability of contouring on MV scans (conformity index, 0.83) was similar to that previously seen for kilo voltage (kV) scans.^118,119^ With the 10 patients combined, the median position of the (axial) centre of the rectum was close to its position on the kV planning scan. This finding is interesting in that it endorses the idea that the planning scan provides an acceptable estimate of position for a group of patients, for the purposes of RT planning, but it also shows that there are differences for individuals who are not accounted for.

#### Variation in rectal dose

Several studies have confirmed differences between planned and delivered doses to the rectum in prostate RT.^112–114,120,121^ Based on weekly cone beam CT scans, analysis suggests that the majority of patients (60%^121^ to 75%^112^) have worse rectal DVHs than shown on the treatment plan. Hatton et al^121^ showed that in 12 patients with prostate cancer, the average *V*_40Gy_ for the rectum was worse in all 12 and the *V*_70Gy_ was worse in 9.

These studies have provided early data to support the notion that *D*_A_ is different from planned dose to the rectum in some patients. A major impediment to further progress is the need for an automated system to contour the rectum, or at an even more sophisticated level, to track the voxels of the rectum from day to day, both to calculate *D*_A_ in a timely fashion and to do so for a significant number of patients.

Using the manual contours for the 10 patients mentioned above, the daily rectal dose to produce *D*_A_ was recalculated.^122^ The mean difference in equivalent uniform dose (EUD) was −2.7 Gy (5%) (median, −2.7 Gy), the minus sign indicating a reduction in rectal dose. This represents a 5% mean dose reduction compared with the planned dose. A reduction in EUD was seen in 7 of the 10 patients. The largest dose reduction seen was −10.2 Gy (−17%). Using *D*_50%_ of the rectum, the mean dose difference was −3.3 Gy, or −8% (median, −2.0 Gy, −6%), while the largest difference seen was −17 Gy (−38%).

Using our data, if dose escalation was undertaken in the seven patients in whom the rectal EUD *D*_A_ was less than the planned dose, with doses increased up to the mean EUD, this would allow a mean dose increase of just over 5 Gy. This represents 7.4% of 74 Gy and gives an estimate of the increase in TCP (represented by biochemical progression-free survival) of 5.2%, with some patients having a higher probability and some less (maximum, 9.9%; minimum, 1.8%). Although this relates only to our 10-patient cohort, it illustrates the principle of using *D*_A_ to refine the individualisation of RT.

**Figure 7.**
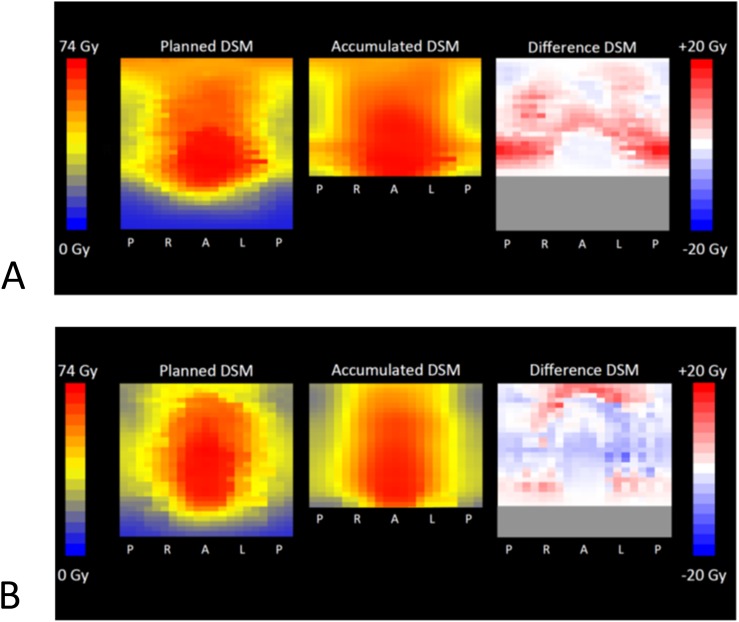
Dose–surface maps (DSMs) for Patient A, with the highest accumulated equivalent uniform dose (EUD) compared with planned (+5.3 Gy) of the 10 patients and for Patient B with the lowest accumulated EUD compared with planned (−10.2 Gy). The rectum was considered a cylinder, and daily delivered dose was sampled at a set of equally spaced points on each MV slice. The cylinder was then “cut” at the point where a vertical line from the centroid of each outline crossed the posterior edge and unfolded. The DSMs were summed over all the fractions, based on the superior–inferior positions of each image corrected for the shifts applied at treatment. Results are shown as accumulated DSMs; planned DSMs are shown for comparison. The difference DSM represents the difference for each pixel between accumulated and planned dose. Since the length of the MV CT image set was less than that of the rectum, the difference DSM is shorter (shown in grey). Although Patient A had a median *D*_A_ of 1.7 Gy higher than planned, areas of the superior rectum received doses of up to 2.8 Gy less than planned. Patient B had a median *D*_A_ of −0.8 Gy compared with that planned; in this case, inferior and superior rectum received up to 13.9 Gy more than planned. A, anterior; L, left; P, posterior; R, right. Reproduced from Scaife et al^122^ with permission from the British Institute of Radiology.

This DVH approach to analysis of *D*_A_ has the advantage of producing quantitative data that can be compared with the equivalent planning data. However, it gives no spatial information about dose distribution; this is vital if links between physical dose difference and toxicity are to be uncovered. The summing of DVHs from different fractions can give misleading results, since the positions of the high dose region will vary from fraction to fraction, and the lack of spatial information means that potentially no tissue actually receives the highest dose shown in the accumulated DVH. Therefore, we have used algorithms based on those described by Murray et al^123^ and Buettner et al^106^ to produce accumulated rectal dose–surface maps (DSMs) for the 10 patients. These show that, despite a median difference in dose (*D*_A_ minus planned dose) of only −0.06 Gy at the pixel level, the range of dose differences was between −17.5 Gy and +20.1 Gy. These differences affected areas of the rectal surface away from the prostate, the region where set-up is verified, and are consistent with data from cone beam imaging in three patients.^123^ DSMs for 2 of the 10 patients in our study are shown in Figure 7.

## COMBINING NORMAL TISSUE SENSITIVITY AND ACCUMULATED DOSE (*D*_A_) DATA FOR RADIOTHERAPY INDIVIDUALISATION

Considering the mean of our DSM data as an estimate of the population mean, then 4 of the 10 patients had rectal dose reductions greater than this. Coincidentally, one approach to radiosensitivity testing might identify 40% of patients as having more resistant normal tissue. Combining the two approaches might identify 16% of patients (*i.e. *1 in 6) who might tolerate even greater dose escalation to the tumour without increasing their NTCP. Table 1 illustrates the simple approach of combining the biological sensitivity information with the physical accumulated dose information to provide individualisation from both strategies. In total, 48% (*i.e.* almost 1 in 2) might benefit from the combination, with either dose escalation, suggesting improved tumour cure or reduced toxicity or both.

**Table 1.**
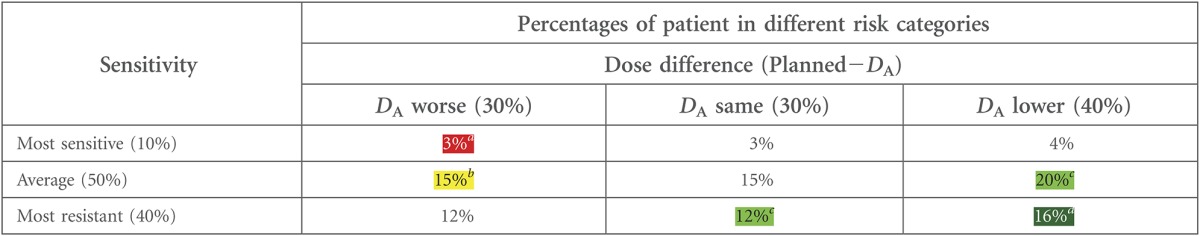
Percentages of patients falling into different categories of risk and the potential for dose alteration, based on both biological radiosensitivity and physical accumulated dose (*D*_A_)

Note that a further analysis of *D*_A_ would be required in order to confirm that *D*_A_ for the individual is actually lower than the population dose limits rather than simply lower than the individual planned dose. The colours are designed to indicate possible treatment strategy alterations: light green indicates modest dose escalation (for either biological or physical rationale) and dark green represents higher dose escalation (resulting from the use of both rationales). Yellow might indicate patients at risk of worse complications for either biological or physical reasons, and red would suggest substantial risk of toxicity. Exactly what might be performed for the “at risk” patients is less clear.

For radiosensitive patients, the initial assumption might be to manage all 10% differently. However, if the strategy was to dose reduce and then follow RT with some additional treatment, potentially those with lower *D*_A_ could receive standard RT. In principle, *D*_A_ could be assessed halfway through the course. If deteriorating, hyperfractionation could be introduced to abrogate toxicity for suitable tumours (*e.g.* head and neck cancer), since this has been shown to reduce toxicity.^124^ This strategy might be unsatisfactory for prostate cancer if the *α* : *β* ratio is confirmed as a low figure; results from the CHHiP trial will be important in clarifying this.^34^ Other therapeutic strategies might also be possible.^54^ For those in categories to the lower right of the table, the possibility of dose escalation is of interest, modest in scale for 32% and higher for 16% of patients. Although the escalation may be limited, small differences are worthwhile, as noted above, and might be applicable to almost half the population.

## CONCLUSIONS

There is clear potential value in predicting an individual's risk of toxicity following RT. The optimal approach is likely to involve both biological and physical data, and combining the two presents synergistic opportunities.

Although there is considerable promise for biological predictive tests, there are currently none that are clinically usable. All such studies require large numbers of patients, with established toxicity phenotypes, and the logistical challenges are considerable. GWASs are revealing polymorphisms with definite links to toxicity risk, and it is likely that the next few years will see an increasing number of these.^88,93,94^ In addition, the GWAS approach is likely to reveal much more about the underlying biology of radiation normal tissue effects, which is an additional valuable goal.

There are challenges too in measuring physical determinants of RT toxicity. Estimates of *D*_A_ can already be made, using manual contouring on image guidance scans. The challenge here is to be able to automate the process in order to upscale the calculations, to allow real-time estimates to be made so that alterations to treatment become possible. The data presented represent only an example of what might be achievable; significant technical challenges remain before this could be introduced into routine care.

Different strategies for manipulating normal tissue dose and sensitivity in order to achieve both reduced toxicity and increased tumour control will be of considerable benefit to patients requiring RT. The development work for these two strategies, as well as many others, is ongoing, and patients should become the beneficiaries from the application of this integrated multidisciplinary approach.

## ACKNOWLEDGMENTS

We are grateful to our colleagues in the VoxTox Programme team: Mrs Amy Bates, Mr Karl Harrison, Professor Andy M Parker, Dr Marina Romanchikova, Mr Michael Simmons and Dr Michael Sutcliffe.
